# New diagnosis of cancer in mild and moderate/severe traumatic brain injury patients in a 12-year population-based study

**DOI:** 10.1186/s12885-022-09416-4

**Published:** 2022-03-18

**Authors:** Chung-Che Lu, Tee-Tau Eric Nyam, Chung-Han Ho, Jinn-Rung Kuo, Chung-Ching Chio, Jhi-Joung Wang, Che-Chuan Wang

**Affiliations:** 1 Departments of Neurosurgery, Chi-Mei Medical Center, 901 Chung Hwa Road, Yung Kang, Tainan, Taiwan; 2 Departments of Medical Research, Chi-Mei Medical Center, Tainan, Taiwan; 3 Department of Information Management, Southern Taiwan University of Science and Technology, Tainan, Taiwan; 4 Cancer Center, Wan Fang Hospital, Taipei Medical University, Taipei, Taiwan; 5 Center for General Education, Southern Taiwan University of Science and Technology, Tainan, Taiwan

**Keywords:** Traumatic brain injury (TBI), Mild TBI, Moderate/severe TBI, Cancer, Mortality, Population-based

## Abstract

**Background:**

Traumatic brain injury (TBI) has been reported as a risk factor for brain cancer development. However, the magnitude of the impact of TBI on systemic cancer development has not been clarified.

**Methods:**

A retrospective longitudinal cohort study was conducted using the Taiwan Longitudinal Health Insurance Database between January 2000 and December 2011. A total of 35,306 patients were initially enrolled, and 14,795 patients with mild TBI and 14,795 patients with moderate/severe TBI were matched using the National Health Insurance Research Database in Taiwan. The Cox proportional hazard regression model was used to estimate the hazard ratio (HR) of TBI adjusted for potential confounding factors.

**Results:**

After matching, the results showed that patients with moderate/severe TBI had a high mortality rate (17.7% vs. 10.4%) and shorter time interval from TBI to death (mean 3.6 years vs. 5.8 years). No differences were observed in cancer incidence (4.1% vs. 4.1%) or risk factors for mortality between mild and moderate/severe TBI patients. However, patients aged between 46 and 55 years, female patients, and patients with pre-existing renal disease had a significant higher cancer incidence risk in moderate/severe TBI compared with mild TBI patients. The top 15 most common cancers showed that mild TBI patients had a higher percentage of head and neck cancer. The overall mortality rate in all TBI patients diagnosed with cancer was about 50%, and the cancer-specific mortality is approximately 85% in death of TBI patients with cancer.

**Conclusions:**

We concluded that the incidence risk of a new cancer diagnosis and mortality risk of TBI patients with cancer between the mild TBI and moderate/severe TBI patients were not significantly different.

**Supplementary Information:**

The online version contains supplementary material available at 10.1186/s12885-022-09416-4.

## Introduction

An estimated 18.1 million new cancer cases and 9.6 million cancer deaths occurred globally in 2018 [1]. Carcinogenesis is a multifactorial and multiple-step process that proceeds through the sequential stages of initiation, promotion, and progression [2]. In addition to genetic factors [3] and the dosage of radiation exposure [4], chronic inflammation [5, 6] and neuroendocrine [7] and metabolic deregulation [3] may promote the development of cancer.

Traumatic brain injury (TBI) is a public health problem. The most common causes of TBI include falls, vehicle collisions and violence [8]. It is estimated that sixty-nine million individuals suffer TBI from all causes worldwide each year [9]. TBI is a disease process that may persist for months to years post-injury [10]. In addition to the local effects of a TBI on the brain, TBI may lead to systemic effects by activating neuroinflammation, followed by non-neurological dysfunctions in the cardiovascular system, lungs, liver, gastrointestinal tract, kidneys and neuroendocrine system [11]. These effects have a similar pathophysiology as carcinogenesis [5–7], especially the local effect of TBI, which may be related to brain tumors, including benign [12] and malignant tumors [13, 14].

A remote effect on increased incidence and risk for new-onset systemic cancer among patients has been reported [15, 16]. Based on a nationwide population study in Taiwan, Ho et al. showed that the risk of developing cancer is higher in patients with chronic obstructive pulmonary disease (COPD) than in patients without COPD [15]. Lin et al. also demonstrated that dialysis is associated with a higher risk of cancer in patients with end-stage renal disease [16]. Therefore, we would like to observe remote effects that originate from the brain on increasing the risk of a new systemic cancer diagnosis.

In Taiwan [17], the mortality rate of patients with cancer remains high and cancer is a leading cause of mortality for all populations [18]. A novel finding in our recent study indicated that TBI patients had a 1.27-fold higher risk for a new diagnosis of systemic cancer than those without TBI, and our TBI patients had greater frequencies of cancer in the head and neck structures [19]. Based on the concept that injury severity is a critical outcome predictor following TBI [20, 21], whether the severity of TBI affects systemic malignant cancer development is worth investigating. Therefore, this time-series study was conducted to further estimate the differences in cancer risk between mild TBI patients and moderate/severe TBI patients.

Thus, the aims of this study were to compare the cancer risk, major cancer types, and mortality associations between patients with mild TBI and patients with moderate/severe TBI during a follow-up period. We hypothesized that the severity of TBI may affect the incidence risk of a new cancer diagnosis and mortality risk in cancer patients with TBI. Our results provide a foundation for facilitating the prevention of new-onset systemic cancer after TBI.

## Methods

### Data sources and research design

Taiwan’s National Health Insurance Research Database (NHIRD), which is extensively used by published studies, was used in this study. All patients with records from 2001 to 2011 were assessed from inpatient medical beneficiaries, a subset database of the NHIRD, to identify those with TBI. The detailed diagnostic information from the clinical visits of each insured subject was based on the clinical modification of the International Classification of Diseases, Ninth Revision, Clinical Modification (ICD-9-CM) code. A retrospective longitudinal cohort study was conducted using the Taiwan Longitudinal Health Insurance Database between January 2000 and December 2011. The NHIRD consists of unidentifiable secondary data that have been released to the public for research; thus, the requirement for ethics approval was waived by the Institutional Review Board at Chi-Mei Medical Center (10,607-E01). All methods were carried out in accordance with the relevant guidelines and regulations. The requirement for informed consent was waived due to the retrospective nature of the study.

### Patient selection and definition

**Fig. 1:**
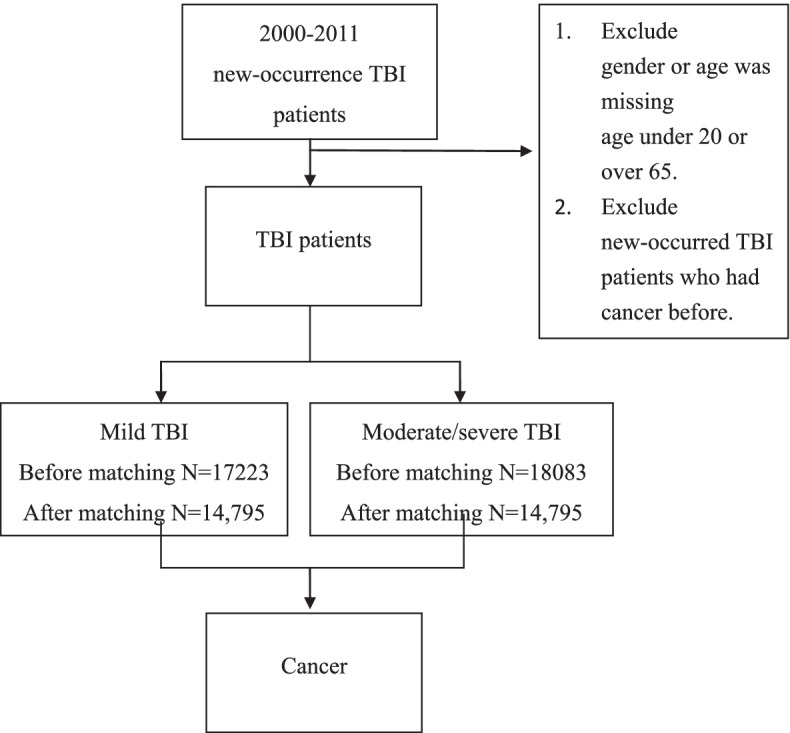
Flow chart of the participants throughout the study

Figure 1 shows a flowchart of the study scheme. The case group included patients with a new occurrence of moderate/severe TBI that occurred between 2000 and 2011. The control group included patients with a new occurrence of mild TBI that occurred between 2000 and 2011. TBI was defined using ICD-9-CM codes 800.0–801.9, 803.0–804.9, 850.0–854.1, or 959.01, and the following codes were classified as mild TBI: ICD-9-CM with the first four digits of 800.0, 800.5, 801.0,801.5, 803.0, 803.5, 804.0, 804.5, 850.0, 850.1, 850.5, or 850.9 (with a fifth digit of 0, 1, 2, 6, 9, or missing digits) or 854.0 (with a fifth digit of 1, 2, 6, 9, or missing digits) [22]. Moderate/severe TBI was defined as all moderate/severe TBI cases, and all diagnoses that were used were from inpatients. We excluded data if information for sex or age was missing, if the patient’s age was younger than 20 years or older than 65 years, and if the patients with a new occurrence of TBI had cancer previously.

Age was defined as the first date of TBI occurrence minus the patient’s birthday. The ICD-9 code definitions of comorbidities included the following: hypertension (HTN) 401–405; hyperlipidemia 272; diabetes mellitus (DM) 250; renal disease 582, 583, 585, 586, and 588; coronary artery disease 410, 411, 412, 413, and 414; and stroke 430, 431, 432, 433, 434, 435, 436, 437, and 438. Information on cancer diagnosis was obtained from the Taiwan Cancer Registry, and information on death was obtained from the Cause of Death Data.

To reduce the potential confounding effects of age and sex on cancer incidence among TBI patients, a propensity score matching approach with age and sex was used in this study. The SAS macro “*%OneToManyMTCH*” was used to calculate the propensity score with the nearest-neighbor matching algorithm [23].

### Measurements

The primary outcome in this study was cancer. Cancer status was based on the Registry of the Catastrophic Illness database. According to guidelines from the Ministry of Health and Welfare, when a patient is diagnosed with cancer, after more than two physicians conduct a formal review, the patient can apply for a catastrophic illness certificate. In most cases, patients who receive a cancer diagnosis undergo regularly scheduled treatment, such as surgery, radiotherapy, and chemotherapy. In addition, different cancer types were also indicated on the catastrophic illness certificate, including lung cancer, liver cancer, colorectal cancer, prostate cancer, and esophageal cancer in male patients and lung cancer, breast cancer, colorectal cancer, liver cancer, and stomach cancer in female patients. Subgroup analyses according to age, sex, and comorbidities also assessed the risk of overall cancer and different cancer types.

### Statistical analysis

All analyses were performed using Statistical Analysis System (SAS) statistical software (version 9.3; SAS Institute, Inc., Cary, NC). Continuous variables are presented as the means with standard deviations (SDs), and categorical variables are shown as counts and percentages. Pearson’s chi-square test or Fisher’s exact test for categorical variables and Student’s t test or the Wilcoxon rank-sum test for continuous variables were used to compare the baseline distribution between the patients with TBI and the compared cohorts. The Kaplan–Meier method was used to plot the trend of cancer incidence with the log-rank test to compare the risk of cancer incidence between the two groups. The Cox proportional regression model was applied to estimate the hazard ratio of cancer adjusted by potential confounding factors. A *p* value < 0.05 was considered significant. The Kaplan–Meier curves were plotted using STATA (version 12; Stata Corp., College Station, TX).

## Results

### Demographic characteristics

Figure 1 shows the flow chart of this study. The demographic and comorbidity information is presented in Table 1. Before matching, of the 35,306 patients with TBI, 17,223 mild TBI patients and 18,023 moderate/severe TBI patients were enrolled in this study. The overall sex ratio was 2:1 for males to females, and the mild TBI group had more females than males (42. 6% for mild TBI vs. 33. 5% for moderate/severe TBI, *p* value < 0.0001). Most patients with TBI were aged 20 to 35 years, and the mild TBI group was younger than the moderate/severe TBI group. After matching by sex and age, the comorbidities of HTN, DM, renal disease and stroke were significantly different between the patients with mild TBI and those with moderate/severe TBI (*p* < 0.0001) after adjustment. Patients with moderate/severe TBI presented with higher mortality (17.4% vs. 10.4%, *p* < 0. 0001) and shorter average time from TBI to death (3.6 years vs. 5.8 years, *p* < . 0001) than patients with mild TBI. The cancer incidence was not significantly different between the patients with mild TBI and patients with moderate/severe TBI (Table 1).

**Table 1:** Demographic and clinical information of mild TBI and moderate/severe TBI patients

	**Before matching**			**After matching**		
**Variables**	**Mild TBI** **(*N* = 17,223)**	**Moderate/severe TBI** **(*N* = 18,083)**	***p* value**	**Mild TBI** **(*N* = 14,975)**	**Moderate/severe TBI** **(*N* = 14,975)**	***p* value**
**Sex**						
Male	9891 (57.43)	12,028 (66.52)	< 0.0001	9227(61.62)	9254(61.80)	0.7482
Female	7332 (42.57)	6055 (33.45)		5748(38.38)	5721(38.20)	
**Age**						
20–35	7283 (42.29)	6567 (36.32)	< 0.0001	6067(40.51)	6078(40.59)	0.9781
36–45	3816 (22.16)	3753 (20.75)		3181(21.24)	3152(21.05)	
46–55	3644 (21.16)	4183 (23.13)		3327(22.22)	3346(22.34)	
55–65	2480 (14.40)	3580 (19.80)		2400(16.03)	2399(16.02)	
**Comorbidity**						
HTN	1719 (9.98)	2496 (13.80)	< 0.0001	1594(10.64)	1835(12.25)	< 0.0001
DM	1084 (6.29)	1532 (8.47)	< 0.0001	1000(6.68)	1122(7.49)	0.0060
Hyperlipidemia	1096 (6.38)	1335 (7.38)	0.0002	1001(6.68)	987(6.59)	0.7452
CAD	583 (3.39)	749 (4.14)	0.0002	533(3.56)	579(3.87)	0.1598
Renal disease	188 (1.09)	386 (2.13)	< 0.0001	165(1.10)	279(1.86)	< 0.0001
Stroke	389 (2.26)	1003 (5.55)	< 0.0001	363(2.42)	742(4.95)	< 0.0001
**Follow-up period (years)**	10.64 ± 3.40	9.11 ± 3.16	0.0021	9.62 ± 3.66	8.90 ± 4.27	< 0.0001
**Death**						
Yes	1740 (10.10)	3376 (18.67)	< 0.0001	1559(10.41)	2654(17.72)	< 0.0001
No	15,483 (89.90)	14,707 (81.33)		13,416(89.59)	12,321(82.28)	
**Time to death (year)**	5.93 ± 3.96	3.37 ± 3.77	< 0.0001	5.82 ± 3.93	3.56 ± 3.92	< 0.0001
**Cancer**						
Yes	710 (4.12)	753 (4.16)	0.8441	608(4.06)	610(4.07)	0.9533
No	16,513 (95.88)	17,330 (95.84)		14,367(95.94)	14,365(95.93)	
**Time to cancer (year)** **Median (IQR)**	5.08 (2.66–7.91)	4.75 (2.33–7.58)	0.0539	4.75(2.46–7.66)	4.96(2.58–7.92)	0.5378

*TBI* traumatic brain injury, *HTN* hypertension, *DM* diabetes mellitus, *CAD* coronary artery disease, *IQR* interquartile range

### Outcome analysis

**Fig. 2:**
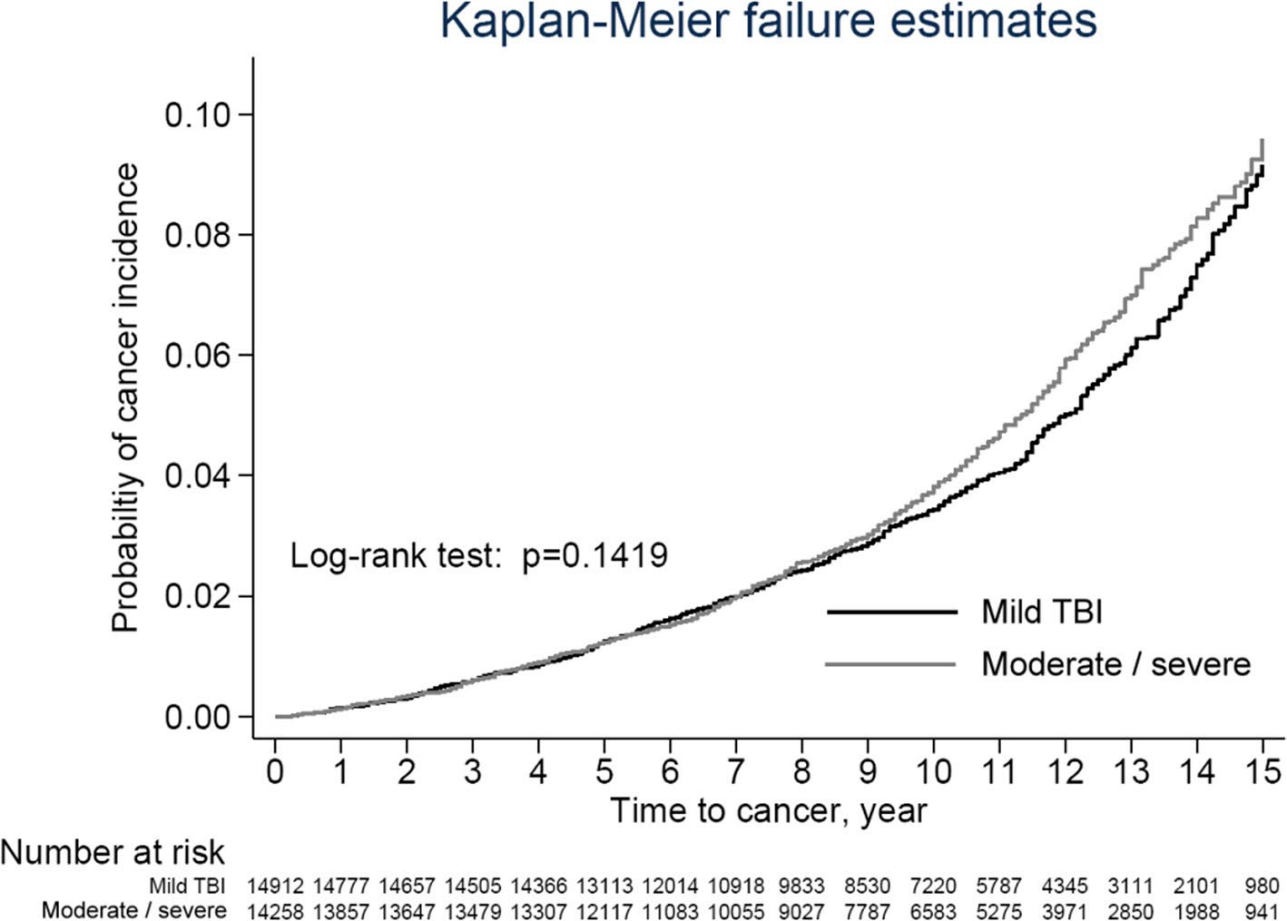
Kaplan–Meier plot of cancer development among the moderate/severe TBI patients and mild TBI patients

Figure 2 presents the cancer development risk trend between the patients with moderate/severe TBI and the patients with mild TBI after matching (log-rank test *p* = 0.1419). Table 2 presents the risk of incident cancer for the overall and stratified groups between the patients with moderate/severe TBI and the patients with mild TBI. Patients with moderate/severe TBI had a 1.1-fold higher risk with no statistically significant of a new cancer diagnosis than patients with mild TBI (95% C.I.: 1.1(1.0–1.2); *p* = 0.0750). In the stratified analysis, patients with moderate/severe TBI exhibited a statistically significant higher risk of a new cancer diagnosis than patients with mild TBI among females (HR: 1.3; 95% C.I.: 1.0–1.4; *p* value = 0.0219), patients aged 46 to 55 years (HR: 1.3; 95% C.I.: 1.1–1.6; *p* value = 0.0069), and patients with a history of renal diseases (HR: 2.5; 95% C.I.: 1.1–5.6; *p* value = 0.0273).

**Table 2:** Incidence risk of cancer for patients with moderate/severe TBI compared with patients with mild TBI

	**Before matching**				**After matching**			
**Variables**	**Mild TBI**	**Moderate/** **severe TBI**	Adjusted* HR **(95% CI)**	***p* value**	**Mild TBI**	**Moderate/severe TBI**	Adjusted* HR **(95% CI)**	***p* value**
	**Cancer (%)**	**Cancer (%)**			**Cancer (%)**	**Cancer (%)**		
**Overall**	710 (4.1)	753 (4.2)	1.14(1.03–1.27)	0.0127	608(4.1)	610(4.1)	1.11(0.99–1.24)	0.0751
**Sex**								
Male	436 (4.4)	506 (4.2)	1.11(0.97–1.26)	0.1241	406(4.4)	377(4.1)	1.04(0.90–1.19)	0.6173
Female	274 (3.7)	247 (4.1)	1.21(1.02–1.44)	0.0316	202(3.5)	233(4.1)	1.25(1.03–1.51)	0.0219
**Age**								
20–35	92 (1.3)	70 (1.1)	1.08(0.79–1.48)	0.6143	67 (1.1)	60(1.0)	1.19(0.84–1.68)	0.3393
36–45	167 (4.4)	130 (3.5)	0.98(0.78–1.24)	0.8703	133(4.2)	108(3.4)	0.98(0.76–1.26)	0.8518
46–55	235 (6.4)	288 (6.9)	1.35(1.13–1.60)	0.0008	211(6.3)	252(7.5)	1.29(1.07–1.55)	0.0069
55–65	216 (8.7)	265 (7.4)	1.06(0.89–1.28)	0.5118	197(8.2)	190(7.9)	0.97(0.80–1.19)	0.7947
**Comorbidity**								
HTN	142 (8.3)	159 (6.4)	0.98(0.78–1.23)	0.8546	129(8.1)	118(6.4)	0.92(0.71–1.18)	0.5035
DM	85 (7.8)	91 (5.9)	0.92(0.68–1.24)	0.5605	80(8)	68(6.1)	0.92(0.67–1.28)	0.6340
Hyperlipidemia	67 (6.1)	70 (5.2)	1.06(0.75–1.50)	0.7403	57(5.7)	56(5.7)	1.13(0.78–1.64)	0.5106
CAD	29 (5.0)	51 (6.8)	1.51(0.95–2.41)	0.0839	28(5.3)	41(7.1)	1.48(0.91–2.40)	0.1151
Renal disease	11 (5.9)	31 (8.0)	2.17(1.08–4.36)	0.0303	8(4.8)	27(9.7)	2.49(1.11–5.58)	0.0273
Stroke	25 (6.4)	57 (5.7)	1.10(0.68–1.76)	0.7254	23(6.3)	36(4.9)	1.01(0.59–1.71)	0.9787

*TBI* traumatic brain injury, *HR* hazard ratio, *CI* confidence interval, *HTN* hypertension, *DM* diabetes mellitus, *CAD* coronary artery disease

Tables 3 and 4 show the top 15 cancer types in the mild TBI and moderate/severe TBI groups, respectively. Malignant neoplasms of the liver and intrahepatic bile ducts were predominant in both groups. The mild TBI patients had a higher percentage of head and neck cancer (22.7%) than the moderate/severe TBI patients (19.7%).

**Table 3:** Top 15 cancer types of the mild TBI patients before and after matching

	**Before matching**		**After matching**	
**Rank**	**Malignant cancer type**	**No. of patients (%)**	**Malignant cancer type**	**No. of patients (%)**
1	Liver and intrahepatic bile ducts	151(21.27)	Liver and intrahepatic bile ducts	118(19.41)
2	Trachea, bronchus, and lung	76(10.70)	Trachea, bronchus, and lung	58(9.54)
3	Female breast	72(10.14)	Female breast	52(8.55)
4	Cervix uteri	58(8.17)	Cervix uteri	32(5.26)
5	Colon	45(6.34)	Colon	32(5.26)
6	Other and unspecified parts of the mouth	44(6.20)	Other and unspecified parts of the mouth	31(5.10)
7	Rectum, rectosigmoid junction, and anus	36(5.07)	Rectum, rectosigmoid junction, and anus	30(4.93)
8	Esophagus	33(4.65)	Esophagus	26(4.28)
9	Tongue	28(3.94)	Tongue	24(3.95)
10	Hypopharynx	23(3.24)	Stomach	19(3.13)
11	Nasopharynx	20(2.82)	Hypopharynx	16(2.63)
12	Stomach	20(2.82)	Nasopharynx	14(2.30)
13	Skin	20(2.82)	Skin	14(2.30)
14	Thyroid gland	19(2.68)	Oropharynx	14(2.30)
15	Oropharynx	18(2.54)	Thyroid gland	13(2.14)
16	Others	47(6.62)	Others	115(18.91)

**Table 4:** Top 15 cancer types of the moderate/severe TBI patients before and after matching

	**Before matching**		**After matching**	
**Rank**	**Malignant cancer type**	**No. of patients (%)**	**Malignant cancer type**	**No. of patients (%)**
1	Liver and intrahepatic bile ducts	140(18.59)	Liver and intrahepatic bile ducts	95(15.57)
2	Female breast	67(8.9)	Female breast	66(10.82)
3	Trachea, bronchus, and lung	65(8.63)	Trachea, bronchus, and lung	47(7.7)
4	Colon	58(7.7)	Colon	40(6.56)
5	Esophagus	48(6.37)	Rectum, rectosigmoid junction, and anus	34(5.57)
6	Other and unspecified parts of the mouth	42(5.58)	Esophagus	33(5.41)
7	Rectum, rectosigmoid junction, and anus	42(5.58)	Cervix uteri	29(4.75)
8	Cervix uteri	36(4.78)	Other and unspecified parts of the mouth	29(4.75)
9	Tongue	29(3.85)	Tongue	24(3.93)
10	Hypopharynx	28(3.72)	Hypopharynx	20(3.28)
11	Prostate	27(3.59)	Stomach	20(3.28)
12	Stomach	25(3.32)	Prostate	15(2.46)
13	Thyroid gland	18(2.39)	Thyroid gland	14(2.3)
14	Bladder	17(2.26)	Skin	12(1.97)
15	Body of the uterus	14(1.86)	Body of the uterus	12(1.97)
16	Others	97(12.88)	Others	120(19.67)

**Fig. 3:**
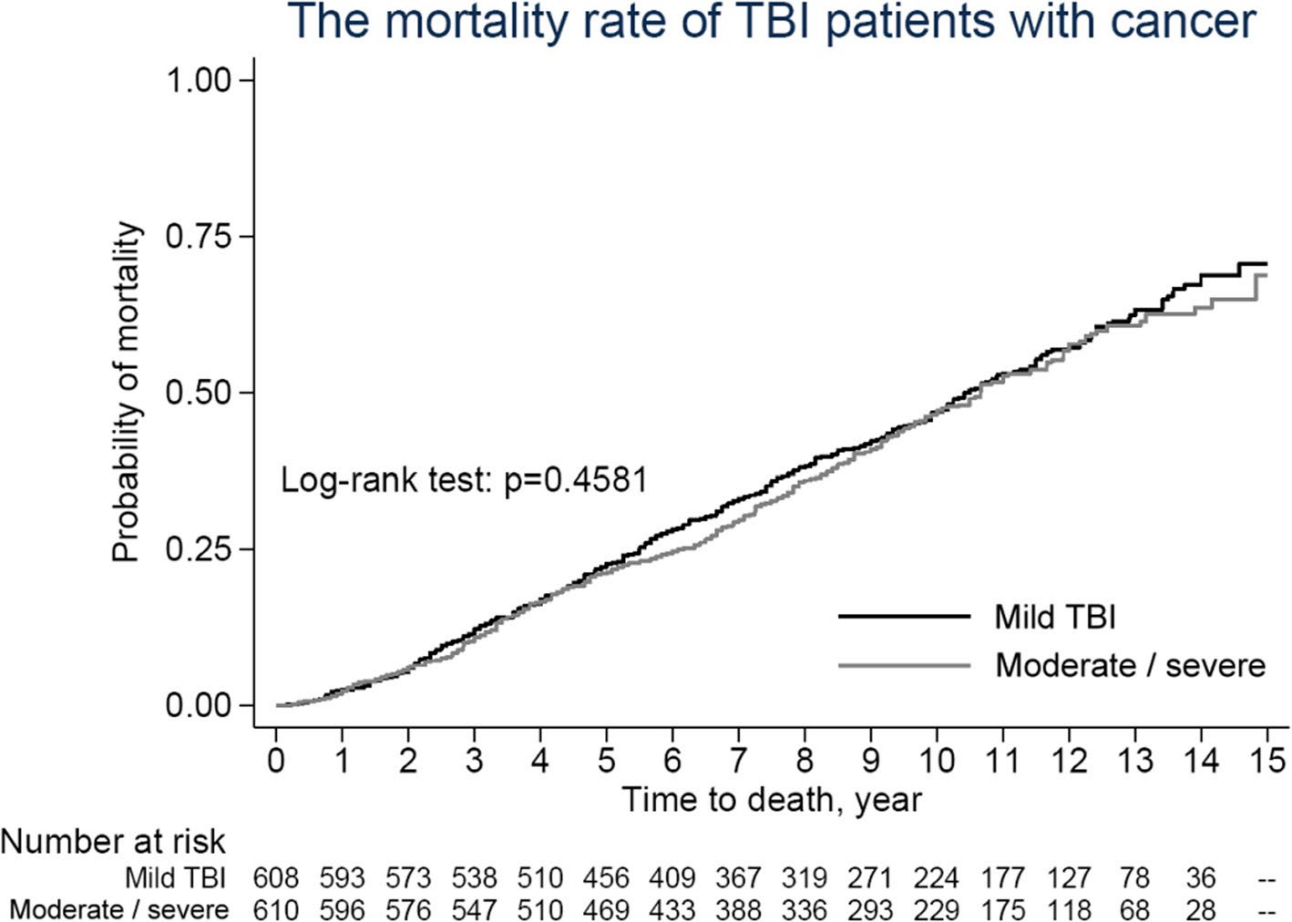
The trends of mortality among the moderate/severe TBI patients and mild TBI cancer patients

Figure 3 indicates the trends in the overall mortality rate in patients with cancer between the moderate/severe TBI and mild TBI groups (log-rank test *p* = 0.4581), and Table 5 presents the overall and stratified analyses of mortality risk among patients with cancer between the patients with moderate/severe TBI and patients with mild TBI after matching. However, no significant difference in mortality risk was found in patients with TBI and cancer.

**Table 5:** Overall and stratified analysis of mortality risk among patients with cancer between the patients with moderate/severe TBI and patients with mild TBI

	**Before matching**		**Before matching**	
Variables	Adjusted* HR (95% CI)	***p* value**	Adjusted* HR (95% CI)	***p* value**
**Overall**	1.01 (0.88–1.17)	0.8500	0.99(0.85–1.16)	0.9291
**Sex**				
Male	1.03 (0.87–1.21)	0.7436	1.00(0.84–1.19)	0.9746
Female	1.01 (0.74–1.39)	0.9294	0.98(0.69–1.38)	0.9011
**Age**				
20–35	1.16 (0.68–1.96)	0.5935	1.23(0.67–2.26)	0.5025
36–45	1.10 (0.80–1.51)	0.5612	1.01(0.71–1.44)	0.9554
46–55	0.93 (0.73–1.19)	0.5732	0.90(0.70–1.17)	0.4398
55–65	1.07 (0.84–1.36)	0.5800	1.07(0.82–1.40)	0.6206
**Comorbidity**				
HTN	1.16 (0.84–1.59)	0.3636	1.09(0.78–1.54)	0.6135
DM	0.86 (0.57–1.29)	0.4663	0.79(0.51–1.21)	0.2777
Hyperlipidemia	1.38 (0.79–2.39)	0.2573	1.03(0.57–1.86)	0.9204
CAD	1.60 (0.82–3.11)	0.1661	1.52(0.78–2.94)	0.2184
Renal disease	1.14 (0.37–3.53)	0.8237	1.72(0.54–5.46)	0.3602
Stroke	1.00 (0.53–1.89)	0.9925	1.14(0.58–2.23)	0.7146

*TBI* traumatic brain injury, *HR* hazard ratio, *HTN* hypertension, *DM* diabetes mellitus, *CAD* coronary artery disease

For all study subjects, 48.5% (710/1463) of the TBI patients with cancer died. In addition, the all cancer-specific mortality of all death was 87.5% (322/368) in patients with mild TBI, and 85.8% (345/402) in patients with moderate/severe TBI (Table 6). Therefore, there was no significant difference in the overall mortality rate and cancer-specific mortality rate for all deaths between the patients with moderate/severe TBI and patients with mild TBI (Table 6). In the subgroup analysis, nearly 12.5% of the patients died from competing causes in patients with mild TBI. Infection (9 deaths, 29.0%), diabetes mellitus (5 deaths, 16.1%), and cardiovascular disease (4 deaths, 12.9%) accounted for 58% of all competing causes [Appendix A]. Additionally, 14.2% of the patients with moderate/severe TBI died from competing causes. Cerebrovascular disease (7 deaths, 20.1%), infection (6 deaths, 17.6%), and accidents, including self-harm (5 deaths, 14.7%), accounted for 52.4% of all competing causes [Appendix B].

**Table 6:** Difference in cancer-specific mortality for all deaths between the patients with moderate/severe TBI and patients with mild TBI

	Mild TBI with cancer (*N* = 710)	Moderate/severe TBI with cancer (*N* = 753)	*p* value
Overall mortality	368(51.83)	402(53.39)	0.5515
cancer-specific mortality of all deaths	322(87.50)	345(85.82)	0.4942

## Discussion

According to our review of the literature, this study is the first to demonstrate the association between the severity of TBI and cancer development based on a national population-based database. In this study, we added new information to the field of oncology: in a comparison of patients with mild TBI and matched patients with moderate/severe TBI, no differences were observed in cancer incidence and the risk factors for mortality. Although moderate/severe TBI patients had a higher mortality rate and shorter interval from TBI to death, moderate/severe TBI patients aged 46–55 years old, were females, and had pre-existing renal disease show higher incidence risk of cancer than mild TBI patients. Mild TBI patients had a higher percentage of head and neck cancer.

Before matching, we found that patients with moderate/severe TBI were older and had a greater burden of all evaluated comorbidities (Table 1). We believe that the theoretical basis for an association between TBI and systemic cancer risk is tentative, and as such, we should consider the very real possibility that the reported associations are due to residual confounding related to older age and poorer health in the moderate/severe TBI group. Therefore, we consider that the HR for cancer in individuals with moderate/severe TBI could be attenuated after adjustment for these factors. This consideration was confirmed after matching, as the results show (Table 2).

In this study, during a mean follow-up period of 9.6 years for mild TBI patients and 8.9 years for moderate/severe TBI patients, the patients with moderate/severe TBI presented with a higher mortality (17.7% vs. 10.4%) and shorter time from TBI to death (mean 3.6 vs. 5.8 years). We also found that patients with mild TBI had a mortality rate of 10.4% within a mean time of 5.8 years. This information on the life expectancy of patients with mild or moderate/severe TBI can inform treatment decisions, assist in the utilization of limited medical resources, and provide plans for ongoing medical needs and lifelong planning.

Carcinogenesis is a multiple-step process that includes initiation, promotion, and progression [2]. Therefore, it takes a long time to develop malignancy; for example, clinical development period estimates range from 20–25 years for colon cancer [24]. However, the starting time point of malignant development is difficult to determine; therefore, we set the beginning of the observation period to 2000. Consistent with our previous study, the mean time from TBI to cancer diagnosis was 4.8 years among mild TBI patients and 5.0 years among moderate/severe TBI patients; these durations are shorter than that among the general population, which is approximately 5.9 years [19]. Because it is hard to understand that cancer can occur within such a short period of time after a traumatic brain injury, we believe that cancer may also be a risk factor for brain injury, given that the cause and effect relationship needs to be clarified. This issue needs to be clarified in the future.

In the current study, we found that moderate/severe TBI increased the cancer incidence risk by 1.1-fold with borderline significance (*p* = 0.0751) compared with mild TBI. This result implied that the severity of TBI may play a potential role in cancer incidence risk. Philip et al. indicated that inflammation, especially TNF-α production, is a tumor promoter in the context of cancer induction [25]. Ohana et al. demonstrated that the transcription factors of the p53, HIF-1a and c-Myc oncogenes play a role in inflammation and in the development of glioblastomas after TBI [14]. In our previous study, we demonstrated that TBI may activate local and systemic TNF-α production [26–28]; therefore, we speculate that if the inflammatory response continues and becomes chronic in moderate/severe TBI patients, it will persistently activate transcription factors and increase the probability of malignant transformation. We suggest that neuroinflammation may play a role in the development of systemic cancer, although we did not study neuroinflammation in the current report. However, the role of the neuroinflammatory cascade in the association between moderate/severe TBI and cancer warrants further investigation.

In the general population, females have a lower cancer risk than males [29, 30], and most cancer incidence increases with age [31, 32]. McCarthy et al.et al. indicated that among women, menopause and hormone replacement therapy were risk factors for cancer development [29]. In our current study, after matching and adjustment, we provided new information that female patients and patients aged 46 to 55 years had a higher cancer risk incidence in moderate/severe TBI patients (Table 2). These results implied that injury severity would affect the cancer incidence risk in these specific groups. This information reminds physicians to be careful of the long-term effects of moderate/severe TBI on cancer development. We consider these data to lay the foundation for future TBI and cancer research.

Previous studies have reported significantly higher new-onset cancer risk in end-stage renal disease patients than in the general population [32, 33]; the pathologies included renal cell carcinoma and urothelial cell carcinoma [33]. In the current study, we further found that TBI patients with renal disease had a higher new-diagnosis cancer risk than non-renal disease patients. However, the underlying mechanisms of increased cancer risk in patients with TBI stratified by renal disease are not well known, and the relationship warrants further investigation. Furthermore, in mild and moderate/severe TBI patients, whether the incidence of cancer is higher in patients with renal disease, not related to TBI but only related to the risk of cancer from renal disease, needs to be clarified in the future.

When analyzing patients with mild TBI and with moderate/severe TBI (Tables 3 and 4), head and neck cancer included mouth, esophageal, tongue, hypopharynx, nasopharynx, oropharynx, and thyroid gland cancers. There was no significant difference between the mild TBI and moderate/severe TBI patients (22.7% vs. 19.7%, *p* = 0.1960). This result implies that the severity of TBI does not affect cancer type. We compared the top 15 cancers with those in 2014 in Taiwan and found that hepatocellular and bile duct carcinomas were the most frequent cancers in patients with mild (19.4%) and moderate/severe (15.6%) TBI in our study; 11,358 new cases of hepatobiliary tumors were diagnosed in 2014, and hepatobiliary tumors accounted for 11.0% of the total cancer in Taiwan [34]. However, there was no significant difference between the mild TBI and moderate/severe TBI patients (19.4% vs. 15.6%, *p* = 0.08). This result also implies that the severity of TBI does not affect cancer type.

In the current study, we also found that patients had higher frequencies of head and neck cancer in both mild and moderate/severe TBI patients compared to the general population in Taiwan [30]. The detailed mechanisms are not well understood. We posit that patients with TBI had a greater chance of receiving brain computer tomography radiation exposure, which may have played a role in cancer development [35]. Berrington et al. estimated that the mean lifetime cancer risk is 0.04% to 0.09% per head CT [36]. Brenner and Hall demonstrated that as many as 1.5% to 2.0% of all cancers in the United States may be attributable to radiation from CT studies [37]. Sale et al. also showed that the incidence of radiation-induced head and neck cancer was 1% [38]. Because patients with TBI frequently receive large numbers of radiographic examinations during hospitalization [39], they are more likely to develop head and neck tumors. Other possible mechanisms are related to menopause and hormone replacement therapy as a risk for head and neck and esophageal cancer [39]. Because tobacco use and alcohol use are major risk factors for head and neck cancer [40, 41], it is necessary to compare smoking and alcohol consumption in the mild TBI group to the moderate/severe TBI group in the future.

On average, the death of TBI patients with cancer was 86.7% of cancer-specific causes. However, infection, diabetes mellitus, and cardiovascular disease accounted for 58.0% of all causes of death beyond malignancy in patients with mild TBI [Appendix A]. Cerebrovascular disease, infection, and accidents, including self-harm, accounted for 52.4% of all causes of death beyond malignancy in the patients with moderate/severe TBI [Appendix B]. Therefore, we consider identifying patients at high risk for mortality and developing preventive interventions to be necessary, especially in moderate/severe TBI patients with a risk of self-harm. Another topic for further research is to clarify the effects of TBI severity on each cancer cause of mortality.

In Taiwan, mortality from cancer remains the leading cause of death among all the population [17, 18]. Based on our results, we found 4.1% of each TBI patients with mild or moderate/severe developed cancer during the follow/up. Because of a subset of patients with more severe forms of TBI may lapse in their cancer treatment and patients symptomatic from mild TBI experience further gaps in evaluating for cancer symptoms and/or reaching systems of care for a new cancer diagnosis. Therefore, we recommend clinicians should be particularly aware that if a patient with a brain injury during follow-up has symptoms of suspected cancer such as loses weight, a lump on palpation and prolonged coughing, blood in the stool, etc., they should arrange tests to rule out the possibility of cancer.

The strengths of our study include the use of Taiwan’s NHIRD, which is a longitudinal nationwide database with a large sample size and a long follow-up period to address a topic about which relatively little is known. We combined the NHIRD with the Taiwan Cancer Registry and the Cause of Death Data, making it possible to estimate the cancer incidence risk among TBI patients.

Some limitations should be considered when interpreting these data. First, the NHIRD did not provide information on the severity of cancer, the use of drugs such as hormone replacement, the dosage of radiation exposure, lifestyle patterns, smoking, alcohol intake, or occupational hazards that may affect tumor progression and prognosis. Second, the radiographic injury location [42] and surgical interventions [43] were not considered during matching or controlled, which is a limitation when considering the severity of injury. Third, hepatobiliary cancers and breast cancers were rarely observed in this study, and a history of hepatobiliary and breast diseases should be considered as a comorbidity. Therefore, the existence of various confounding factors cannot be denied. Finally, both head trauma and cancer are frequent, and tangible proof of an association is required. These issues should be clarified in the future.

## Conclusions

No significant difference was observed in the incidence risk of a new diagnosis of cancer and mortality risk in TBI patients with cancer between mild TBI and moderate/severe TBI patients. In all cases of the death of TBI patients with cancer, more than 85% patients died of cancer.

## Supplementary Information

**Additional file 1: Appendix A.** Cause of death in mild TBI patients after having cancer (*N*=368).

**Additional file 2: Appendix B.** Cause of death in moderate/severe TBI patients after having cancer (*N*=402).

## Data Availability

The datasets used and/or analyzed during the current study are available from the corresponding author upon reasonable request.

## Abbreviations

TBI: Traumatic brain injury
HTN: Hypertension
DM: Diabetes mellitus
CAD: Coronary artery disease
IQR: Interquartile range
HR: Hazard ratio
CI: Confidence interval
