# Supplementary material for: New diagnosis of cancer in mild and moderate/severe traumatic brain injury patients in a 12-year population-based study
Source: BMC Cancer. 2022 Mar 18;22:291. doi: 10.1186/s12885-022-09416-4 (PMC8933911; doi:10.1186/s12885-022-09416-4)
Supplement: Supplementary file 1 — Additional file 1: Appendix A. Cause of death in mild TBI patients after having cancer (N=368). [file 12885_2022_9416_MOESM1_ESM.docx]

**Appendix A. Cause of death in mild TBI patients after having cancer (N=368)**

| **ICD9** | **Disease** | **Number of individuals** | **Percentage** |
| --- | --- | --- | --- |
| 155 | Malignant neoplasm of liver and intrahepatic bile ducts | 83 | 22.6 |
| 162 | Malignant neoplasm of trachea, bronchus, and lung | 49 | 13.3 |
| 150 | Malignant neoplasm of esophagus | 29 | 7.9 |
| 145 | Malignant neoplasm of other and unspecified parts of mouth | 22 | 6.0 |
| 153 | Malignant neoplasm of colon | 16 | 4.4 |
| 151 | Malignant neoplasm of stomach | 12 | 3.3 |
| 174 | Malignant neoplasm of female breast | 12 | 3.3 |
| 571 | Chronic liver disease and cirrhosis | 11 | 3.0 |
| 141 | Malignant neoplasm of tongue | 9 | 2.5 |
| 146 | Malignant neoplasm of oropharynx | 7 | 1.9 |
| 147 | Malignant neoplasm of nasopharynx | 7 | 1.9 |
| 148 | Malignant neoplasm of hypopharynx | 7 | 1.9 |
| 156 | Malignant neoplasm of gallbladder and extrahepatic bile ducts | 7 | 1.9 |
| 157 | Malignant neoplasm of pancreas | 7 | 1.9 |
| 199 | Malignant neoplasm without specification of site | 7 | 1.9 |
| 189 | Malignant neoplasm of kidney and other and unspecified urinary organs | 5 | 1.4 |
| 191 | Malignant neoplasm of brain | 5 | 1.4 |
| 250, E14.9 | Diabetes mellitus | 5 | 1.4 |
| 161 | Malignant neoplasm of larynx | 4 | 1.1 |
| 185 | Malignant neoplasm of prostate | 4 | 1.1 |
| 517 | Lung involvement in conditions classified elsewhere | 4 | 1.1 |
| *C97 | Malignant neoplasms of independent (primary) multiple sites | 4 | 1.1 |
| 159 | Malignant neoplasm of other and ill-defined sites within the digestive organs and peritoneum | 3 | 0.8 |
| 38.9 | Septicaemia, unspecified | 2 | 0.5 |
| 149 | Malignant neoplasm of other and ill-defined sites within the lip, oral cavity, and pharynx | 2 | 0.5 |
| 154 | Malignant neoplasm of rectum, rectosigmoid junction, and anus | 2 | 0.5 |
| 173 | Other malignant neoplasm of skin | 2 | 0.5 |
| 180 | Malignant neoplasm of cervix uteri | 2 | 0.5 |
| 183 | Malignant neoplasm of ovary and other uterine adnexa | 2 | 0.5 |
| 188 | Malignant neoplasm of bladder | 2 | 0.5 |
| 202 | Other malignant neoplasms of lymphoid and histiocytic tissue | 2 | 0.5 |
| 205 | Myeloid leukemia | 2 | 0.5 |
| 486 | Pneumonia, organism unspecified | 2 | 0.5 |
| 730 | Osteomyelitis, periostitis, and other infections involving bone | 2 | 0.5 |
| 158 | Malignant neoplasm of retroperitoneum and peritoneum | 1 | 0.3 |
| 163 | Malignant neoplasm of pleura | 1 | 0.3 |
| 170 | Malignant neoplasm of bone and articular cartilage | 1 | 0.3 |
| 172 | Malignant melanoma of skin | 1 | 0.3 |
| 179 | Malignant neoplasm of uterus, part unspecified | 1 | 0.3 |
| 182 | Malignant neoplasm of body of uterus | 1 | 0.3 |
| 183 | Malignant neoplasm of ovary and other uterine adnexa | 1 | 0.3 |
| 184 | Malignant neoplasm of other and unspecified female genital organs | 1 | 0.3 |
| 193 | Malignant neoplasm of thyroid gland | 1 | 0.3 |
| 208 | Leukemia of unspecified cell type | 1 | 0.3 |
| 431 | Intracerebral hemorrhage | 1 | 0.3 |
| 507 | Pneumonitis due to solids and liquids | 1 | 0.3 |
| 567 | Peritonitis and retroperitoneal infections | 1 | 0.3 |
| 572 | Liver abscess and sequelae of chronic liver disease | 1 | 0.3 |
| 578 | Gastrointestinal hemorrhage | 1 | 0.3 |
| 799 | Other ill-defined and unknown causes of morbidity and mortality | 1 | 0.3 |
| 235 | Neoplasm of uncertain behavior of digestive and respiratory systems | 1 | 0.3 |
| 335 | Anterior horn cell disease | 1 | 0.3 |
| 401 | Essential hypertension | 1 | 0.3 |
| 402 | Hypertensive heart disease | 1 | 0.3 |
| 425 | Cardiomyopathy | 1 | 0.3 |
| 491, 496 | Chronic obstructive pulmonary disease, unspecified | 1 | 0.3 |
| 585, 593 | Chronic renal failure, unspecified | 1 | 0.3 |
| E888 | Other and unspecified fall | 1 | 0.3 |
| *I51.6 | Cardiovascular disease, unspecified | 1 | 0.3 |
| *I64 | Stroke, not specified as haemorrhage or infarction | 1 | 0.3 |
| *N18.0 | End-stage renal disease | 1 | 0.3 |
| ***ICD10** | |  |  |
