# Supplementary material for: New diagnosis of cancer in mild and moderate/severe traumatic brain injury patients in a 12-year population-based study
Source: BMC Cancer. 2022 Mar 18;22:291. doi: 10.1186/s12885-022-09416-4 (PMC8933911; doi:10.1186/s12885-022-09416-4)
Supplement: Supplementary file 2 — Additional file 2: Appendix B. Cause of death in moderate/severe TBI patients after having cancer (N=402). [file 12885_2022_9416_MOESM2_ESM.docx]

| **Appendix B. Cause of death in moderate/severe TBI patients after having cancer (N=402)** | | | | |  |  |
| --- | --- | --- | --- | --- | --- | --- |
| **ICD9** | **Disease** | | **Number of individuals** | **Percentage** |  |  |
| 155 | Malignant neoplasm of liver and intrahepatic bile ducts | | 76 | 18.9 |  |  |
| 162 | Malignant neoplasm of trachea, bronchus, and lung | | 46 | 11.4 |  |  |
| 150 | Malignant neoplasm of esophagus | | 42 | 10.5 |  |  |
| 145 | Malignant neoplasm of other and unspecified parts of mouth | | 20 | 5.0 |  |  |
| 153 | Malignant neoplasm of colon | | 18 | 4.5 |  |  |
| 571 | Chronic liver disease and cirrhosis | | 14 | 3.5 |  |  |
| 148 | Malignant neoplasm of hypopharynx | | 13 | 3.2 |  |  |
| 174. 175 | Malignant neoplasm of breast, unspecified | | 10 | 2.5 |  |  |
| 157 | Malignant neoplasm of pancreas | | 10 | 2.5 |  |  |
| 154, 453 | Malignant neoplasm of rectum, rectosigmoid junction, and anus | | 10 | 2.5 |  |  |
| 151 | Malignant neoplasm of stomach | | 10 | 2.5 |  |  |
| 146 | Malignant neoplasm of oropharynx | | 10 | 2.5 |  |  |
| 141 | Malignant neoplasm of tongue | | 8 | 2.0 |  |  |
| 202 | Unspecified types of non-Hodgkin's lymphoma | | 6 | 1.5 |  |  |
| 185 | Malignant neoplasm of prostate | | 6 | 1.5 |  |  |
| 147 | Malignant neoplasm of nasopharynx | | 6 | 1.5 |  |  |
| 199 | Malignant neoplasm without specification of site | | 5 | 1.2 |  |  |
| 188 | Malignant neoplasm of bladder | | 5 | 1.2 |  |  |
| *C97 | Malignant neoplasms of independent (primary) multiple sites | | 4 | 1.0 |  |  |
| 208 | Leukaemia, unspecified | | 4 | 1.0 |  |  |
| 486 | Pneumonia, organism unspecified | | 4 | 1.0 |  |  |
| 205 | Myeloid leukaemia | | 3 | 0.8 |  |  |
| 161 | Malignant neoplasm of larynx | | 3 | 0.8 |  |  |
| 156 | Malignant neoplasm of gallbladder and extrahepatic bile ducts | | 3 | 0.8 |  |  |
| E14.9 | Diabetes mellitus, Without complications | | 2 | 0.5 |  |  |
| 572 | Hepatic failure, unspecified | | 2 | 0.5 |  |  |
| 414, 429 | Atherosclerotic heart disease | | 2 | 0.5 |  |  |
| 578 | Gastrointestinal hemorrhage | | 2 | 0.5 |  |  |
| 577 | Diseases of pancreas | | 2 | 0.5 |  |  |
| 434 | Occlusion of cerebral arteries | | 2 | 0.5 |  |  |
| 432 | Other and unspecified intracranial hemorrhage | | 2 | 0.5 |  |  |
| 195 | Malignant neoplasm of other and ill-defined sites | | 2 | 0.5 |  |  |
| 191 | Malignant neoplasm of brain | | 2 | 0.5 |  |  |
| 189 | Malignant neoplasm of kidney and other and unspecified urinary organs | | 2 | 0.5 |  |  |
| 183 | Malignant neoplasm of ovary and other uterine adnexa | | 2 | 0.5 |  |  |
| 182 | Malignant neoplasm of body of uterus | | 2 | 0.5 |  |  |
| 180 | Malignant neoplasm of cervix uteri | | 2 | 0.5 |  |  |
| 172 | Malignant melanoma of skin | | 2 | 0.5 |  |  |
| 171 | Malignant neoplasm of connective and other soft tissue | | 2 | 0.5 |  |  |
| 152 | Malignant neoplasm of small intestine, including duodenum | | 2 | 0.5 |  |  |
| 149 | Malignant neoplasm of other and ill-defined sites within the lip, oral cavity, and pharynx | | 2 | 0.5 |  |  |
| 142 | Malignant neoplasm of major salivary glands | | 2 | 0.5 |  |  |
| 038 | Septicemia | | 2 | 0.5 |  |  |
| *Y86 | Sequelae of other accidents | | 1 | 0.3 |  |  |
| *X70 | Intentional self-harm by hanging, strangulation and suffocation | | 1 | 0.3 |  |  |
| *I69.4 | Sequelae of stroke, not specified as haemorrhage or infarction | | 1 | 0.3 |  |  |
| *I21.9 | Acute myocardial infarction, unspecified | | 1 | 0.3 |  |  |
| E957, E959 | Intentional self-harm by jumping from a high place | | 1 | 0.3 |  |  |
| E887, E888, E929 | Unspecified fall | | 1 | 0.3 |  |  |
| *C34.1 | Malignant neoplasm of Upper lobe, bronchus or lung | | 1 | 0.3 |  |  |
| 571A | Lung involvement in conditions classified elsewhere | | 1 | 0.3 |  |  |
| 531, 560 | Other and unspecified intestinal obstruction | | 1 | 0.3 |  |  |
| 345 | Epilepsy, unspecified | | 1 | 0.3 |  |  |
| 203 | Multiple myeloma | | 1 | 0.3 |  |  |
| 198 | Secondary malignant neoplasm of other specified sites | | 1 | 0.3 |  |  |
| 197 | Secondary malignant neoplasm of liver | | 1 | 0.3 |  |  |
| 165 | Malignant neoplasm of Ill-defined sites within the respiratory system | | 1 | 0.3 |  |  |
| 163 | Malignant neoplasm of heart, mediastinum and pleura in Pleura | | 1 | 0.3 |  |  |
| 159 | Malignant neoplasm of Ill-defined sites within the digestive system | | 1 | 0.3 |  |  |
| 968 | Poisoning by other central nervous system depressants and anesthetics | | 1 | 0.3 |  |  |
| 780 | General symptoms | | 1 | 0.3 |  |  |
| 586 | Renal failure, unspecified | | 1 | 0.3 |  |  |
| 570 | Acute and subacute necrosis of liver | | 1 | 0.3 |  |  |
| 437 | Other and ill-defined cerebrovascular disease | | 1 | 0.3 |  |  |
| 426 | Conduction disorders | | 1 | 0.3 |  |  |
| 237 | Neoplasm of uncertain behavior of endocrine glands and nervous system | | 1 | 0.3 |  |  |
| 193 | Malignant neoplasm of thyroid gland | | 1 | 0.3 |  |  |
| 192 | Malignant neoplasm of other and unspecified parts of nervous system | | 1 | 0.3 |  |  |
| 184 | Malignant neoplasm of other and unspecified female genital organs | | 1 | 0.3 |  |  |
| 158 | Malignant neoplasm of retroperitoneum and peritoneum | | 1 | 0.3 |  |  |
| 143 | Malignant neoplasm of gum | | 1 | 0.3 |  |  |
| *** ICD 10** | |  |  | |  |  |
|  | |  |  | |  |  |
